# Unravelling the physics of size-dependent dislocation-mediated plasticity

**DOI:** 10.1038/ncomms6926

**Published:** 2015-01-06

**Authors:** Jaafar A. El-Awady

**Affiliations:** 1 Department of Mechanical Engineering, Johns Hopkins University, 3600 North Charles Street, Baltimore, Maryland 21218, USA

## Abstract

Size-affected dislocation-mediated plasticity is important in a wide range of materials and technologies. Here we develop a generalized size-dependent dislocation-based model that predicts strength as a function of crystal/grain size and the dislocation density. Three-dimensional (3D) discrete dislocation dynamics (DDD) simulations reveal the existence of a well-defined relationship between strength and dislocation microstructure at all length scales for both single crystals and polycrystalline materials. The results predict a transition from dislocation-source strengthening to forest-dominated strengthening at a size-dependent critical dislocation density. It is also shown that the Hall–Petch relationship can be physically interpreted by coupling with an appropriate kinetic equation of the evolution of the dislocation density in polycrystals. The model is shown to be in remarkable agreement with experiments. This work presents a micro-mechanistic framework to predict and interpret strength size-scale effects, and provides an avenue towards performing multiscale simulations without *ad hoc* assumptions.

## Abstract (web summary)

Size-affected dislocation-mediated plasticity is important in a wide range of materials and technologies. Here, El-Awady develops a validated generalized size-dependent dislocation-based model that can predict strength as a function of crystal/grain size and dislocation density.

## Graphical abstract

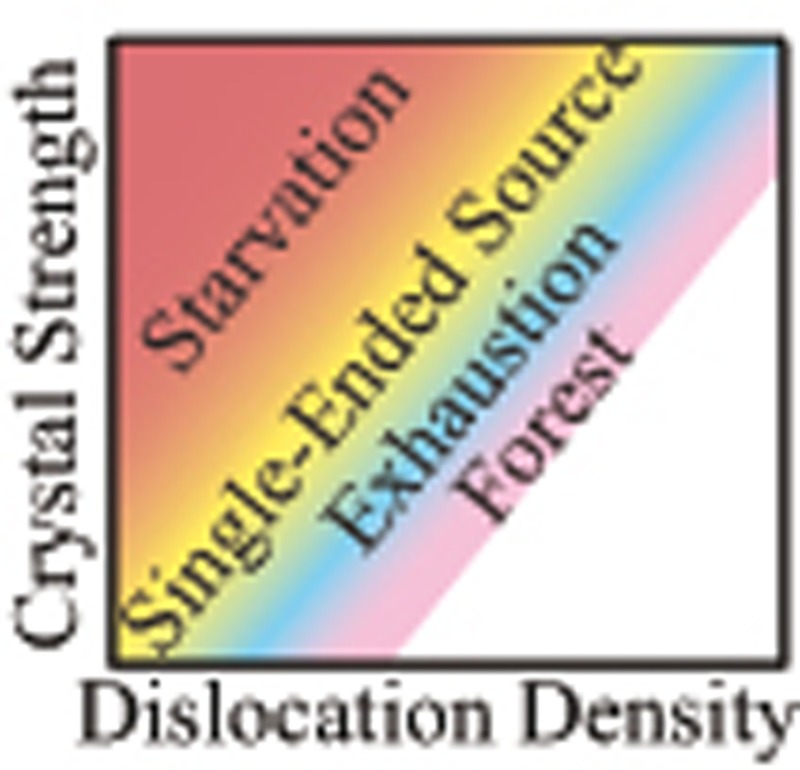

The question of how to explain and predict the effect of size on the properties and response of materials has been at the forefront of mechanics and materials research. Numerous studies have been performed to identify the changes in material properties (for example, thermal^1^, mechanical^2^, magnetic^3^, electric^4^ and so on) as governed by the extrinsic size (for example, crystal external dimensions) or intrinsic size (for example, grain size, distance between precipitates, dislocation cell-structure size and so on), *D*, of the material. An example of an empirically based relationship that is extensively utilized to predict size effects on strength, *σ*, of single and polycrystalline metals is the power-law relationship in the form *σ*=*σ*_0_+*kD*^−*n*^, where *σ*_0_, *k* and *n* are experimentally fitted parameters^5^,^6^,^7^.

In dislocation-mediated plasticity the fundamental building blocks are dislocations, which collectively govern the plastic deformation and damage evolution in metals^8^, semiconductors^9^,^10^, semicrystalline polymers^11^,^12^ and even ceramics under shock loading^13^. It is well established that the strength of bulk crystals increases with increasing dislocation density generally following the well-known Taylor-strengthening power law with an exponent of 0.5 (ref. 14). However, for micron and sub-micron crystals, strength has been observed to increase with decreasing crystal/grain size^2^,^15^,^16^. Furthermore, it is also accepted that the initial dislocation density plays an important role in the strength of micron-sized single crystals, with several simulations and experimental studies showing that bulk like behaviour is recovered at large enough dislocation densities^17^,^18^,^19^,^20^,^21^.

A number of phenomenological relationships were postulated in the literature to account for size effects (for example, refs 22, 23, 24, 25). One of these models, namely the ‘single-ended source model’, was developed to predict size effects in microcrystals^22^. This model is based on computing the probability of finding the maximum size of a single-ended source in a microcrystal of a given diameter and dislocation density. The studies by Zhou *et al*.^23^ and Lee and Nix^26^ showed good agreement between this model and a wide range of experimental data for dislocation densities in the range of 10^12^−10^13^ m^−2^. Furthermore, Phani *et al*.^25^ developed a statistical model based on the random spatial distribution orientation of dislocations in microcrystals. This model was used to explain the strength and scatter in micro-compression and micro-tension experiments of molybdenum alloy fibres. Finally, Gu and Ngan^24^ developed a theoretical model assuming that the yield strength of a microcrystal is determined by Taylor interactions within the initial dislocation network.

Nevertheless, many questions still remain on the effect of dislocation density on the size-dependent response of single crystals and polycrystalline materials. To address this, in the following, we present a study on size-dependent dislocation-mediated plasticity by utilizing a large set of 3D discrete dislocation dynamics (DDD) simulations. These simulations span 2 orders of magnitude in crystal size and 5 orders of magnitude of dislocation density. The results show a correlation between crystal strength and dislocation density for micron and sub-micron crystal sizes, and a minimum crystal strength marked by a transition from dislocation-source strengthening to forest-dominated strengthening at a size-dependent critical dislocation density. These results are validated by a large set of experimental results on micro- and macro-crystals reported previously^19^,^20^,^21^. The developed model is finally shown to agree well with grain size strengthening in polycrystals and provide a microstructurally based understanding of the Hall–Petch relationship.

## Results

### Deformation mechanism map

**Figure 1:**
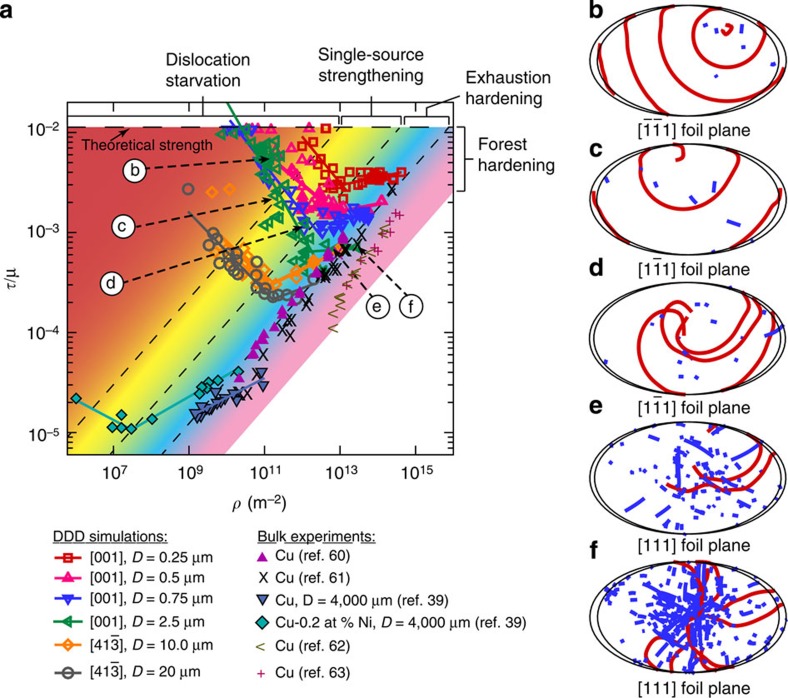
Single-crystal deformation mechanism map. (**a**) Dimensionless-resolved shear strength versus the dislocation density as predicted from DDD simulations. Experimental results from macro-scale Cu crystals^39^,^60^,^61^,^62^,^63^ and Cu-0.2 at%Ni crystals^39^ are also shown. The simulation data for the 10 and 20 μm at *ρ*≥10^11^ m^−2^ are from the study by Rao *et al*.^17^ The deformation mechanism map is shown by coloured contours. (**b**–**f**) show the dislocation microstructure in a thin foil (mimicking a TEM foil) extracted from the middle of the height of several *D*=2.5 μm microcrystals as indicated by arrows in **a**. The dislocations gliding on slip planes parallel to the foil plane are in red, while those intersecting the foil plane are in blue.

**Figure 2:**
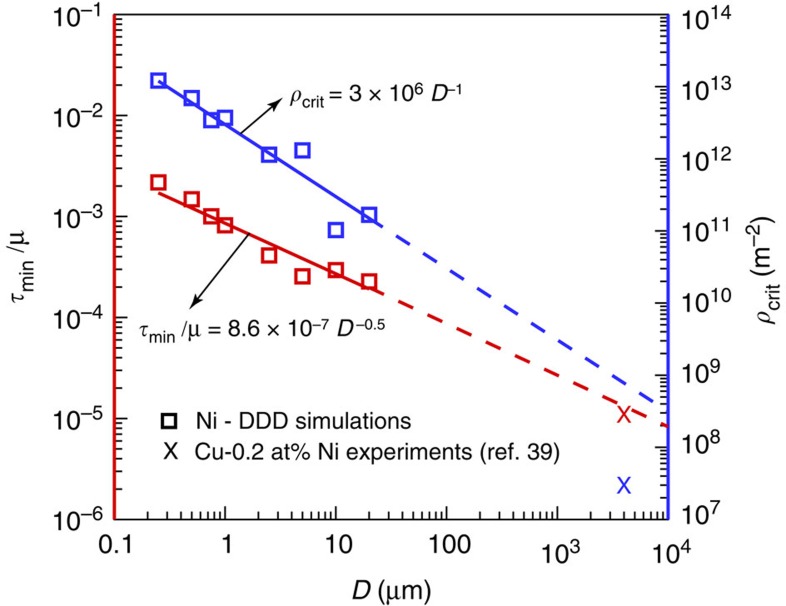
Minimum crystal strength and the critical dislocation density. Minimum-resolved shear strength and critical dislocation density as a function of crystal diameter from Ni micro-scale DDD simulations and Cu-0.2 at%Ni macro-scale experiments^39^. Solid lines show the best power-law fit for the simulations. The dashed lines are extrapolations for larger crystal diameters.

Figure 1 shows a composite plot of the resolved shear strength, *τ*, normalized by the shear modulus, *μ*, versus the initial dislocation density, *ρ*, from 273 DDD simulations of nickel single crystals having diameters in the range 0.25≤*D*≤20.0 μm. The strength is computed between 0.5 and 1.0% strain, and at these strain levels the dislocation density does not increase or decrease more than a factor of 2–3 times from its initial value^23^,^27^,^28^. These results clearly show, for each crystal size, the strength scales with the dislocation density following a power-law relationship of the form *τ*=*ρ*^*n*^, having negative and positive exponents below and above a critical dislocation density, *ρ*_crit_ respectively. Here *ρ*_crit_ is defined as the dislocation density at which the crystal has the lowest attainable strength, *τ*_min_. As shown in Fig. 2, both *ρ*_crit_ and *τ*_min_ are size-dependent properties and are proportional to the inverse of the crystal diameter and inverse of the square root of the crystal diameter, respectively.

In DDD, all dislocations are tracked and their final dispositions are known. Thus, by examining the evolution of the dislocation microstructure from these simulations, four deformation mechanisms can be identified as a function of the crystal size and the initial dislocation density. The inserts in Fig. 1 show the dislocation microstructure at the end of the simulation in thin slices having a thickness of 468 nm. These slices are taken from the middle of the height of five simulated microcrystals, referenced by circled numbers, having *D*=2.5 μm. The coloured regions in Fig. 1 show the range of dominance of each deformation mechanism. The boundaries between these regions are shown as dashed lines and are not definitive due to the unavoidable statistics of source distributions. In the red-shaded region, the governing mechanism is dislocation starvation. In this regime, the dislocation density is small and the number of sources are limited, thus dislocations can easily escape the crystal after activation. This mechanism was first suggested by Greer *et al*.^29^, but only observed experimentally in microcrystals <160 nm in diameter^30^. The absence of a starved crystal in most experiments of specimens having *D*>160 nm can be attributed to the initial dislocation density in these experiments, which is typically reported to be ≥10^13^ m^−2^. It is clear from the mechanism map shown in Fig. 1 that this density is high enough that other deformation mechanisms will dominate. As an example, for starvation to be observed in a *D*=250 nm microcrystal the starting dislocation density should be <10^12^ m^−2^. For larger microcrystals this starting dislocation density needs to be even lower. It is worth noting that in the range of crystal sizes and dislocation densities modelled here, only 9 simulations from the 273 DDD simulation results displayed true starvation behaviour. The second mechanism observed from DDD simulations is the single-source mechanism, dominating in the yellow region (inserts 1, 2 and 3). Here plasticity is governed by the activation of a single source, or a few sparse sources that hardly interact with other dislocations in the crystal^22^,^27^. The third mechanism identified is exhaustion hardening, which is dominant in the light blue-shaded region (insert 4). In this region, the crystal contains relatively high number of dislocation sources that produce a high mobile dislocation density at yield. However, these mobile dislocations exhaust from mutual interactions and the resulting mobile dislocation density is insufficient to sustain flow without increasing the applied stress^17^. Thus, the dislocations that are present do not represent a steady-state mean field of the statistical processes of trapping and source activation that are found for macroscopic flow. Finally, in the purple region the dominant mechanism is forest strengthening (insert 5). Here a high enough dislocation density exists in the crystal and an internal length scale develops, which is in the order of 
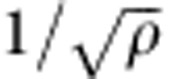
. In this case, the strength is governed by the intrinsic size determined by the steady-state dislocation forest, and is expected, as suggested by experimental observations, to follow the well-known Taylor-strengthening relationship as the initial dislocation density increases^18^,^21^.

It should also be noted that 2.5D DDD^31^,^32^ and 3D DDD simulations^17^,^21^ have both been previously performed to investigate the effect of variations in dislocation density on the microcrystal strength. While those simulations revealed that the strength versus size power-law exponent is affected by the initial dislocation density, they were not able to establish the existence of a strength and density relationship due to the limited range of simulated crystal sizes and starting dislocation densities.

### A generalized size-dependent Taylor-strengthening law

**Figure 3:**
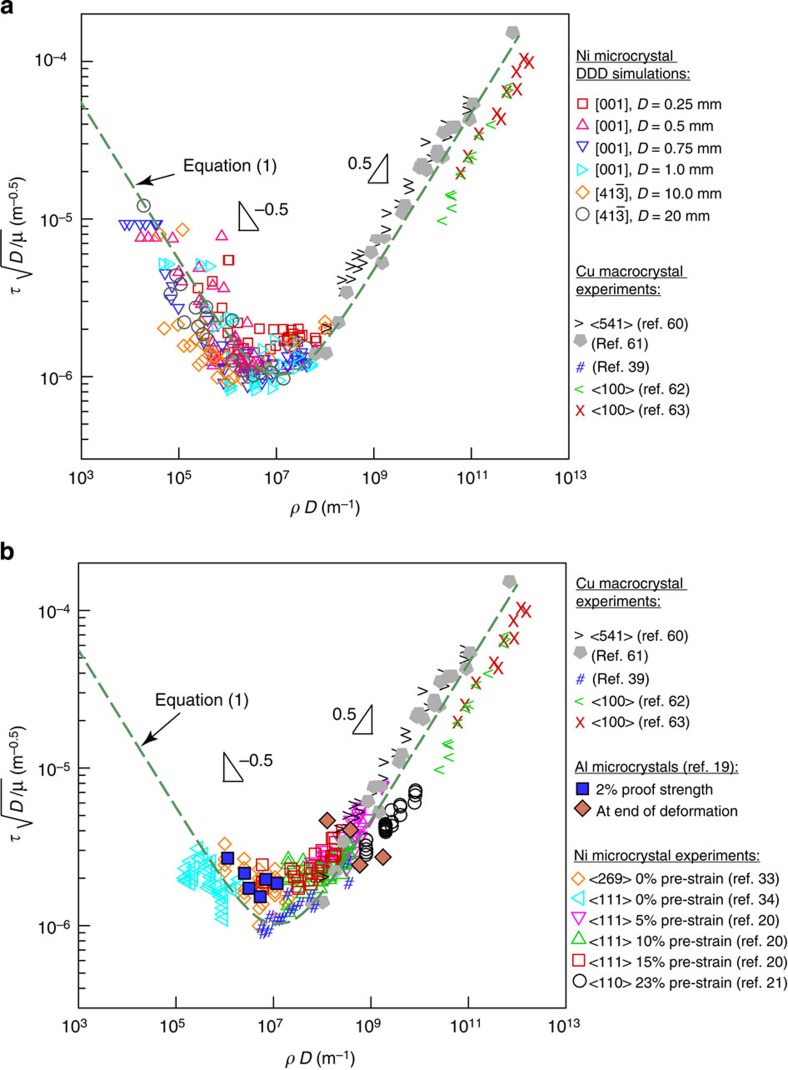
Generalized size-dependent crystal strength. Composite plot of the dimensionless-resolved shear strength multiplied by the square root of the crystal diameter versus the initial dislocation density multiplied by the crystal diameter from: (**a**) DDD simulations of microcrystals in the range 0.25≤*D*≤20 μm and (**b**) experiments on microcrystals in the range 0.2≤*D*≤40 μm (refs 20, 21, 33, 34). Experimental results from Al microcrystals^19^ and Cu bulk single crystals are also shown^39^,^60^,^61^,^62^,^63^.

Both strength and dislocation density at the boundaries between the different mechanism regions shown in Fig. 1 scale with crystal size in a similar manner to *τ*_min_ and *ρ*_crit_. Thus, it is possible to collapse all DDD simulation results onto a single curve by plotting 
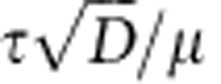
 versus *ρD*, as shown in Fig. 3a. In Fig. 3b, the same scaling relationship for Ni single-crystal experiments ranging in diameter from 0.2 to 40 μm is shown. These microcrystals were focus ion beam (FIB) milled into bulk crystals having initial dislocation densities in the range of 10^12^ to 4 × 10^14^ m^−2^ (refs 20, 21, 33, 34). The experimentally reported strengths are slightly higher on average than those obtained from DDD simulations since they are measured at higher strain levels (that is, between 1 and 5% strain). Similar to the DDD simulations, the pre-existing dislocation density is not expected to change much at these strain levels^35^,^36^, and the effect of FIB damage is negligible in the presence of a pre-existing dislocation network^37^. Nevertheless, the agreement between DDD simulation results and experiments is remarkable. It is worth noting that very few experiments exist at low *ρD* values. Also due to computational limitations it was not possible to simulate crystals having very high values of *ρD*. From Fig. 3, the best fit for the DDD and experimental data is


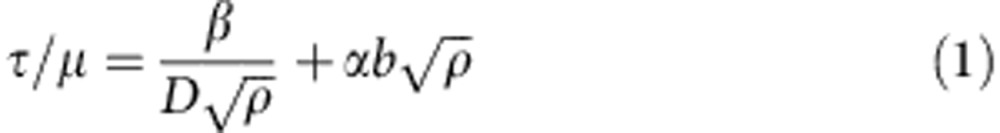


where *β*=1.76 × 10^−3^ and *α*=0.57 are dimensionless constants with *ρ* and *D* in units of m^−2^ and m, respectively. Equation (1) is a generalized size-dependent Taylor-strengthening law. The first term on the right-hand side is the intrinsic substructure length scale, 
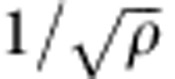
, normalized by the extrinsic length scale of the crystal, *D*. This term is effectively the strength of the weakest dislocation source, which is traditionally expressed in the form *bk*/*λ*, where *k* is a strength coefficient that is typically assumed to be between 0 and 1, and *λ* is the effective (or mean) source length. Thus, the effective source length in the region below the critical dislocation density can be shown to be in the form 
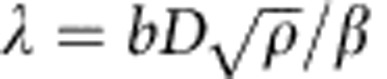
. On the other hand, the second term in equation (1) accounts for forest strengthening, and is proportional to the magnitude of the Burgers vector, *b*, normalized by the intrinsic length scale. Thus, the effective source length in the region above the critical dislocation density can be shown to be in the form 
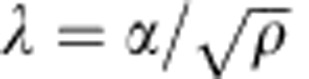
. Further details are discussed in Supplementary Note 1.

It should be noted that the constant *β* could, in general, be a function of the stacking-fault energy, strain, strain rate and temperature. Furthermore, for a very low dislocation density and/or very small crystal size, the limit to equation (1) is the stress at which full dislocations or partial dislocations nucleate from the free surface of the crystal, *γ*/*b*, where *γ* is the stacking-fault energy^22^.

**Figure 4:**
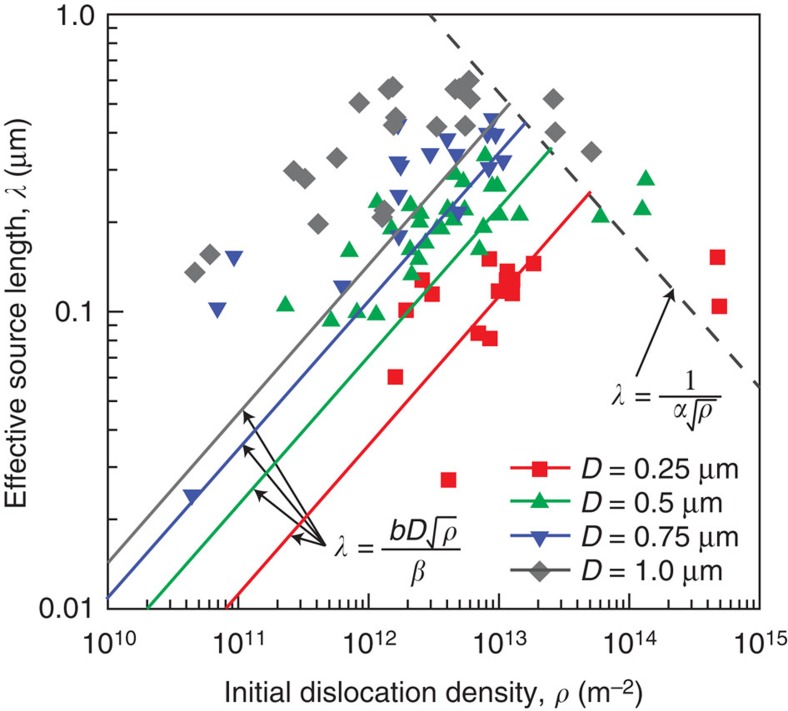
Size-dependent dislocation network length distribution. The effective source length at the onset of plasticity versus the initial dislocation density for four crystal sizes, *D*=0.25, 0.5, 0.75 and 1.0 μm, as computed from DDD simulations (solid symbols). The solid and dashed lines show the predictions from equation (1).

While two qualitative experimental studies of the dislocation microstructure in microcrystals were recently made^35^,^38^, source length characteristics were not identified in those studies. Thus, in the absence of such experimental characterization, the effective source length is computed here from the current DDD simulations. It should be noted that while initially all dislocations in the simulations were randomly distributed with a random length between 0 and *D*, the dislocation network relaxes immediately to accommodate the high local dislocations stress field. Due to these rearrangements, it is expected that the effective dislocation-source length would differ from their initial randomly defined values. Plastic flow then occurs when the stress is high enough to activate the weakest sources available in the crystal. Figure 4 shows the effective source length at the onset of plastic flow versus the initial dislocation density in four different microcrystal sizes, *D*=0.25, 0.5, 0.75 and 1.0 μm as computed from DDD. It is clear from this figure that the DDD predictions agree very well with the effective source length predictions made based on equation (1).

**Figure 5:**
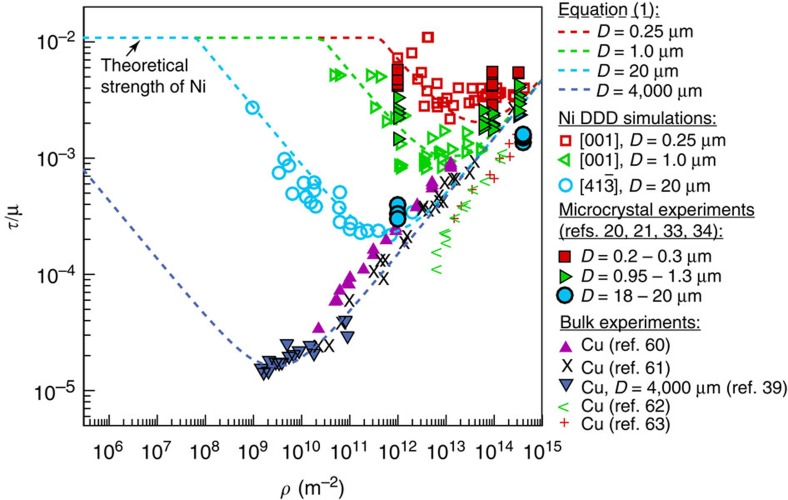
The generalized size-dependent Taylor-strengthening law. Dimensionless-resolved shear strength versus dislocation density as predicted by equation (1) for four different Ni single-crystal sizes. DDD simulations result for Ni and experimental results from Ni microcrystals^20^,^21^,^33^,^34^ and macro-scale Cu single crystals^39^,^60^,^61^,^62^,^63^, are also shown for reference.

The strength versus dislocation density as predicted from equation (1) is plotted in Fig. 5 for different crystal sizes. The DDD results, Ni microcrystal experiments^20^,^21^,^33^,^34^ and Cu bulk-scale experiments^39^ of the same crystal sizes are also shown. This figure shows that unlike when scaled as proposed in Fig. 3, the microcrystal experiments alone are not able to predict the minimum crystal strength or critical dislocation density if plotted on a traditional strength versus dislocation density plot. Finally, the coupled effects of dislocation density and range of studied crystal sizes on the traditional strength versus size power-law exponent are shown in Supplementary Figs 1 and 2, and discussed in Supplementary Note 2.

## Discussion

The above results clearly show the existence of a correlation between the crystal strength and the dislocation density for micron and sub-micron crystal sizes, in agreement with previous experimental and simulation results^16^,^18^,^19^,^20^,^21^. Furthermore, these results show the existence of a size-dependent critical dislocation density that separates between two different deformation mechanisms. While these results show this behaviour at the microscale, the existence of a minimum strength for well-annealed ‘bulk’ copper single crystals was first reported over 45 years ago^40^,^41^,^42^. One of the earliest studies to explain this is the overlooked work of Johnson and Ashby^41^. They postulated that when the dislocation density in a bulk crystal is below some critical value, the probability of finding a dislocation source lying on its slip plane decreases considerably^41^. This dictates that below the critical dislocation density, the dislocation multiplication stress should increase. This analytical model assumes a distribution of dislocation segments forming a network in which dislocations are entangled at randomly distributed three-segment nodes. Thus, hereafter, this model is referred to as the ‘three-segment node model’. Based on this distribution, the probability of finding a dislocation of length *L* or longer while lying on a certain slip plane can be computed. It was shown that the relationship between the crystal strength and the dislocation density is


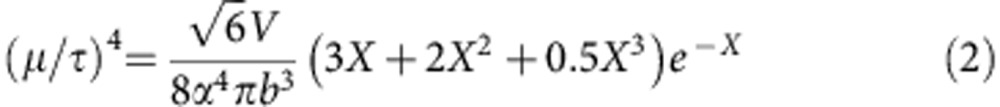


where 
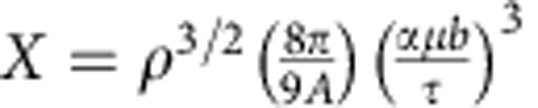
, *V* is the volume of the crystal, and *A*=0.744 is a constant. Equation (2) clearly encompasses the effect of crystal size in the form of the crystal volume. Thus, the minimum strength and critical dislocation density computed from this model are also size dependent. This analytical model predicts that below this critical dislocation density the strength versus dislocation density follows a power-law relationship with an exponent equal to −1.5, while above it the well-accepted Taylor-strengthening relationship, 
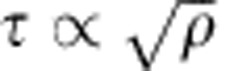
, is recovered. The only assumption made in this model is the character of the dislocation network. Any reasonable dislocation distribution will predict qualitatively a similar response with the minimum strength and critical dislocation density varying by a factor of 3 to 4 (ref. 41). This twofold relationship was observed in well-annealed Cu-0.2 at%Ni a few years later but with exponents equal to 0.2 and −0.2 above and below *ρ*_crit_, respectively^39^. Surprisingly, these experiments and analytical calculations have been neglected as part of the vast ocean of overlooked research. For comparison with the current DDD simulations, these later bulk experimental results are also shown in Fig. 1. A clear agreement between the bulk-scale behaviour and the micro-scale behaviour is observed. The critical strength and the minimum-resolved shear strength for these macrocrystals are also shown in Fig. 2. While the minimum-resolved shear strength matches well the extrapolations from DDD simulations, the critical dislocation density does not show a good agreement. However, comparisons with these Cu-0.2 at%Ni crystals should only by qualitative since they exhibit a combined solid-solution strengthening and dislocation strengthening. The former is not accounted for in the presented DDD simulations or equation (1).

In the context of dislocation density effects, microcrystal experiments were also reported for molybdenum (Mo)^18^,^43^, gold (Au)^44^ and aluminium (Al)^19^ microcrystals. Micro-compression experiments were performed on Mo microcrystals pre-strained to 0, 4 and 11% (ref. 18). The strength was reported to decrease with increasing pre-strain, however, the scatter for the 4% pre-strained microcrystal was huge, which was attributed to variations in the dislocation density from 0 to 2.7 × 10^13^ m^−2^ (ref. 43). Due to the lack of precise dislocation density measurements versus crystal strength in that study, it is difficult to quantitatively compare it with the current results. For Au, micro-compression experiments of 300 nm micropillars fabricated from puck-shaped pillars pre-strained to 6, 13.5, 21, 35 and 41%, were reported^44^. Only the dislocation density in the bare Au-thin film and the 35% pre-strained microcrystal were reported. Thus, other than these two data points, it is difficult to obtain quantitative comparisons with the current DDD simulation results or with the Ni microcrystal experiments. Nevertheless, the authors show that strength continuously decreases with increasing pre-straining with no clear minimum for the crystal strength although the dislocation density reported for the 35% case was ~10^15^ m^−2^. However, by re-examining their transmission electron microscopy micrographs, it could be argued that the dislocation density in the pre-strained puck-shaped pillars is highly non-uniform as compared with the upper half of the pillar, with the density increasing considerably near the MgO interface. Thus, plasticity would be expected to dominate in the lower dislocation density region (the upper half of the microcrystal), since the bottom half would experience large forest hardening due to the higher density of dislocation entanglements in that region. It could be argued that this is supported by the SEM micrographs, showing more dislocation plasticity (surface slip) near the upper half of the crystal versus the bottom half. Finally, micro-compression experiments of pristine and 7% pre-strained Al microcrystals were also performed^19^. The initial dislocation density, the proof strength at 2% strain, as well as the strength and the dislocation density at the end of deformation were all reported. Figure 3 summarizes these experimental results as compared with the current model (equation 1) and Ni microcrystal experiments. It is clear that these Al microcrystal results agree quite well with the model and the Ni data.

Furthermore, a comparison between the single-ended source model^22^, the three-segment node model^41^, and the stochastic model^25^ with micro-scale experiments and equation (1) are shown in Supplementary Fig. 3 and discussed in Supplementary Note 3. It is clear that all these models show an inherent correlation between strength and dislocation density. However, none of these models can capture the universality of the response at the bulk, micron and sub-micron scales, or the correct scaling response below the critical dislocation density. In addition, while these models show a change in response around a critical density, none of them matches the experimental results or the DDD simulations. These models underestimate the critical density and minimum-resolved shear strength relationships and are unable to predict the correlation with crystal size observed from DDD simulations and experiments.

**Figure 6:**
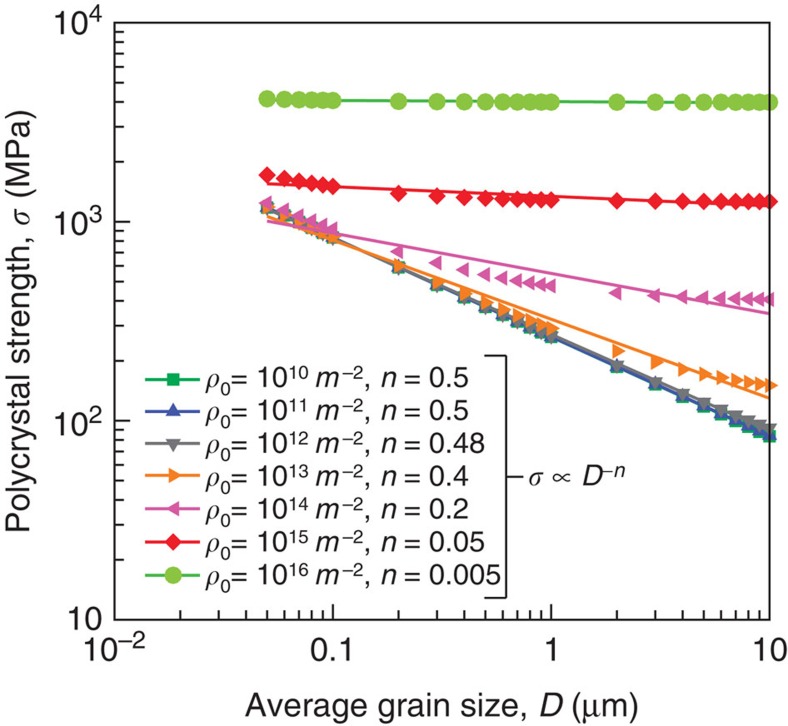
Dislocation density effect on polycrystalline strength. The effect of initial dislocation density on the power-law exponent of the stress versus grain size relationship for nanocrystalline Ni as predicted by equation (1). The symbols are direct predictions from equation (1), and the lines are the best power-law fit (*σ*∝*D*^−*n*^). The power-law exponent at each dislocation density is given in the figure legend.

The question that arises now is whether it is possible to extend equation (1) to predict size effects in polycrystals. One reason for the triumph of the traditional Taylor-strengthening constitutive law over the past eight decades is its effectiveness in predicting the strength of both bulk single crystals and polycrystalline materials^14^. Similarly, for equation (1) to be a generalized size-dependent constitutive law, it should also be applicable to polycrystals. To test this argument, the strength of polycrystals, *σ*, can be deduced from equation (1) by multiplying the shear stress by the ‘Taylor factor’, *M* (ref. 45). In this context, *D* would denote the average grain size of the crystal. The Taylor factor is in the range of 1.73 to 3.67 depending on the condition and texture of the crystal^46^. Figure 6 shows the polycrystalline material strength, from equation (1), as a function of grain size at *M*=2.5 and for different dislocation densities. It is clear that at a constant dislocation density the relationship takes the form *σ*∝*D*^−*n*^ with the power-law exponent in the range 0≤*n*≤0.5. Interestingly, for crystals having an average grain size *D*≤1 μm and having dislocation densities <10^13^ m^−2^, the well-accepted Hall–Petch relationship is recovered (that is, *n*=0.5; (refs 5, 6). As the dislocation density increases, the exponent decreases until the effect of grain size vanishes. This is in qualitative agreement with other experimental and simulation observations^47^,^48^. Note that the strength reported in Fig. 6 is the flow strength for a given dislocation density. No assumption has been made on the evolution of the dislocation density and subsequent hardening, which clearly would be different in single crystals versus polycrystals.

The recovery of the Hall–Petch relationship from equation (1) can be explained as follows. Conrad showed experimentally that the evolution of the dislocation density in any polycrystal is linearly proportional to strain and inversely proportional to the average grain size as


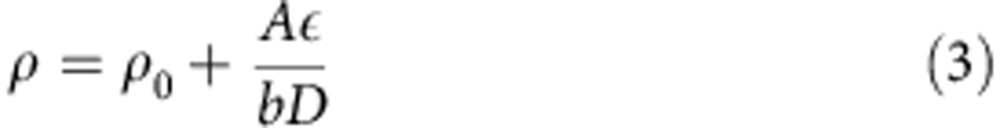


where *ρ*_0_ is the initial dislocation density, 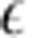 is the stain, and *A* is a constant^49^,^50^,^51^. For polycrystals having an average grain size *D*≤1 μm and an initial dislocation density *ρ*≤10^13^ m^−2^, it can be shown that 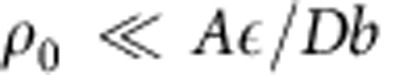. Utilizing this and substituting equation (3) into equation (1) we get


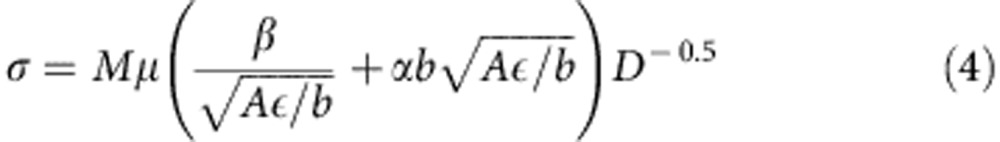


**Figure 7:**
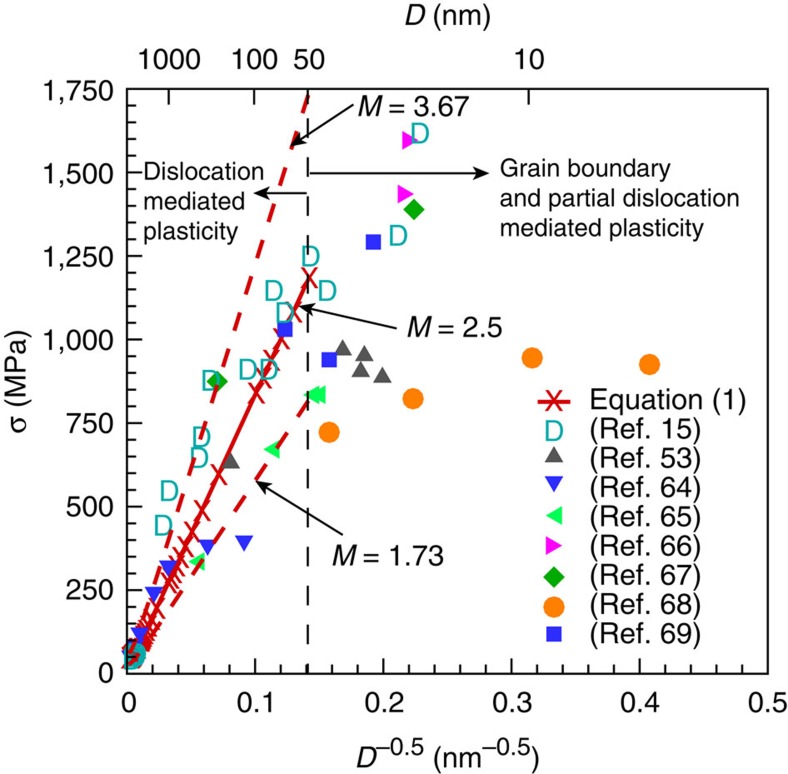
Hall–Petch relationship for Ni. Hall–Petch relationship for nanocrystalline Ni as predicted from equation (1) (solid line for *M*=2.5) and from experiments^15^,^53^,^64^,^65^,^66^,^67^,^68^,^69^. The dashed lines are the lower and upper limits from equation (1) for *M*=1.73 and 3.67, respectively.

Equation (4) suggests that at any strain level, the traditional Hall–Petch relationship holds for small crystal sizes and low initial dislocation densities. For copper, it was shown experimentally that *A*=3.58 (ref. 46), and in the current analysis we assume the same value for nickel. The Hall–Petch plot for polycrystalline Ni as predicted from equations (1) and (3) at yield (that is, 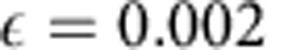) is shown in Fig. 7. It should be noted that it is unrealistic to expect that the initial dislocation density is constant in all grain sizes tested from nanocrystalline to coarse-grained crystals. Instead, the initial dislocation density must be a function of the grain size^52^. Thus, to more reasonably mimic experiments, the initial dislocation density used to predict the red lines in Fig. 7 is assumed to be in the order of 10^12^ m^−2^ for grains *D*>10 μm, and increase inversely with grain size such that *ρ*_0_=10^12^(1+10 × 10^−6^/*D*) μm. Moreover, the solid-curve is for *M*=2.5, and the dashed curves are the limits at *M*=1.73 and *M*=3.67. The results from eight different nanocrystalline experiments are also shown for comparison^15^,^53^. It is clear that the analytical predictions based on equation (1) are in excellent quantitative agreement with the experimental results for an average grain size *D*≥50 nm. For polycrystals with grain sizes *D*<50 nm, the deformation is typically governed by twinning, grain boundary sliding and grain boundary rotations^15^. These deformation mechanisms are not accounted for in equation (1) and are beyond the scope of this study.

It should be mentioned that equation (1) cannot be used alone to predict crystal hardening or softening in single or polycrystalline materials. It must be complimented by a kinetic equation for the evolution of the dislocation density (for example, equation (3)), which will be different for single crystals than for polycrystals. Such coupling will provide an avenue towards performing physics-based simulations at larger scales (for example, crystal plasticity simulations) without *ad hoc* assumptions or nonphysical empirical-based assumptions.

In conclusion, from this study, a size-dependent dislocations-based analytical model was developed using DDD simulations of microcrystals spanning 2 orders of magnitude of crystal sizes and 5 orders of magnitude of dislocation densities. The model is shown to be fully consistent with numerous experimental studies of single crystals and polycrystalline materials. Although this model was based on the total dislocation density, it is possible to re-derive the model based on the density of forest dislocations threading the primary slip plane^54^, or by accounting for the forest interactions between the primary slip system and other secondary systems^55^. Nevertheless, the overall conclusions of this work will not change. Because of the applicability of the model to an extremely large set of crystal/grain sizes (that is, bulk to tens of nanometers) and its portability for predicting the strength of both single and polycrystals, the proposed model is expected to have further applications in constitutive law development and multiscale methods. This model can also be extended to address problems where the high strain rate sensitivity of mobile dislocation density is important.

## Methods

### DDD simulations

In DDD simulations, plasticity is modelled by numerically computing the evolution of dislocation ensembles. These computations can be performed in 2D or 3D computational cells. In 2D DDD simulations, dislocations are modelled as infinite long straight dislocations^56^. On the other hand, 3D DDD methods are based on modelling the full dynamics of dislocation loops in space. All simulations performed here are based on two different 3D DDD techniques. For simulations of microcrystals having diameters *D*<10 μm, the method developed by El-Awady *et al*.^57^ is used. In this approach, dislocations are discretized using curved spline segments^58^, and the image field due to the free surfaces is computed using the boundary element method^57^. Also, screw dislocation cross-slip is accounted for^27^. For simulations involving microcrystals having diameters *D*≥10 μm, an in house modified version of the massively parallel DDD code ‘ParaDis’^59^, developed at the Lawrence Livermore National Laboratory, was used. Here dislocations are discretized as straight line segments. The modifications to the open source version include accounting for dislocation surface interactions and enforcing slip on the appropriate face-centred cubic glide planes^17^. Simulations with this code did not account for dislocation cross-slip.

The material properties used in all simulations are those of Ni single crystals. For simulations involving crystals with *D*<10 μm, the simulation cell is a perfect cylinder having a length to diameter ratio of *L*/*D*=3:1. This mimics the geometry of most micro-compression experiments fabricated by FIB milling^7^. A compressive load is imposed along the axis of the cylindrical cell, which is parallel to the [001] direction, thus insuring multi-slip conditions. For simulations involving microcrystals with *D*≥10 μm the computational cell is cubical with an edge-length equal to *D*. The compressive load imposed in these simulations is along the 
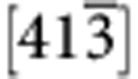
 direction, insuring a nominally single-slip condition. A combined force-controlled and displacement-controlled loading scheme is used in all simulations to mimic experimental studies that do not allow for stress-relaxation^33^.

### Initial dislocation network

The initial dislocation density in the simulations is varied by 5 orders of magnitude from 1 × 10^9^ to 4 × 10^14^ m^−2^. The initial dislocation network is randomly assigned by introducing a distribution of single-ended, double-ended, surface–surface dislocations, or pinning-free circular and dipolar loops. The dislocation-source length is varied based on a two parameter Weibull distribution^27^. It should be noted that our own simulations as well as other published studies show that introducing random pinning points (for example, Frank-Read sources) give quantitatively the same response in terms of size-scale effects as compared with simulations starting from an initially pin-free dislocation network for the same relaxed dislocation densities^28^. For further details about mobility laws, elastic constants and surface boundary conditions, the reader is referred to refs 17, 27, 57.

Finally, the current DDD simulations do not include any criterion to naturally nucleate new dislocations from free surfaces, if the resolved shear stress to activate a pre-existing source is higher than the theoretical stress of the crystal or if all pre-existing dislocations have escaped the crystal. Thus, to address this limitation in the current simulations, if the resolved shear stress exceeds the theoretical strength of the crystal the simulations are aborted and the strength of the crystal is recorded as the theoretical strength of Ni, which is equal to 833 MPa^22^.

## Author contributions

All work presented in this manuscript has been performed by J.A.E.

## Additional information

**How to cite this article:** El-Awady, J. A. Unravelling the physics of size-dependent dislocation-mediated plasticity. *Nat. Commun.* 6:5926 doi: 10.1038/ncomms6926 (2015).

## Supplementary Material

Supplementary Information Supplementary Figures 1-3, Supplementary Notes 1-3, and Supplementary References
