# Supplementary material for: Unravelling the physics of size-dependent dislocation-mediated plasticity
Source: Nat Commun. 2015 Jan 6;6:5926. doi: 10.1038/ncomms6926 (PMC4354076; doi:10.1038/ncomms6926)
Supplement: Supplementary Information — Supplementary Figures 1-3, Supplementary Notes 1-3, and Supplementary References [file ncomms6926-s1.pdf]

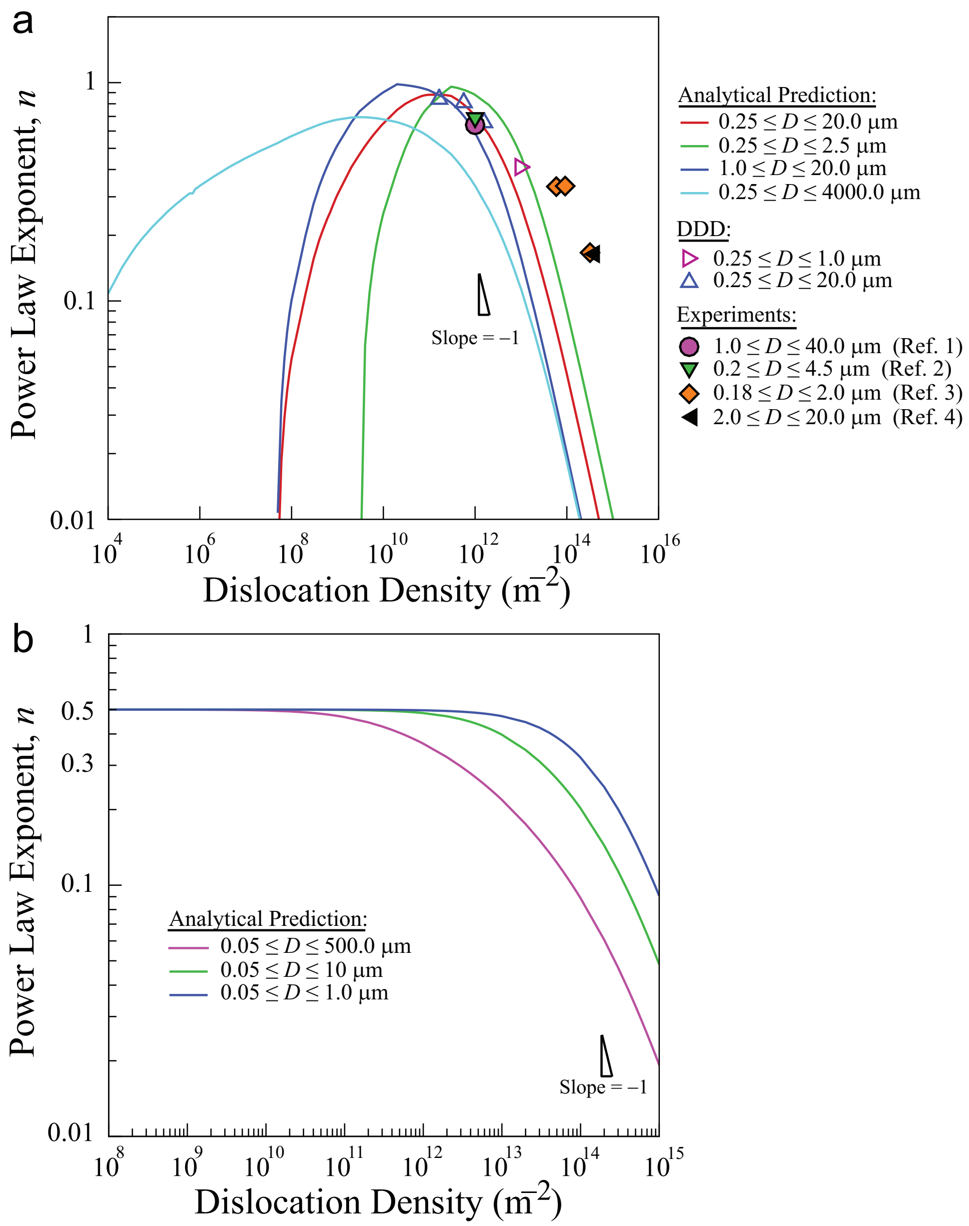

Supplementary Figure 1: **Dislocation density effect on the strength versus size power-law exponent.**

Power-law exponent as a function of dislocation density for (a) Ni single crystals; and (b) Ni polycrystals. Solid lines are the analytical predictions for different ranges of crystal size, while the symbols are predictions from DDD simulations and experiments [1–4].

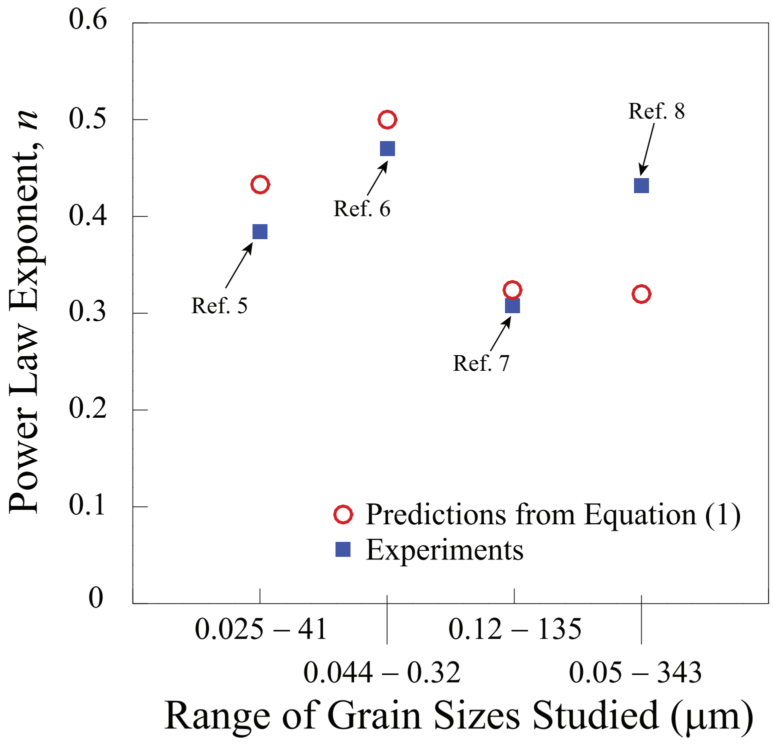

Supplementary Figure 2: **Effect of range of studied grain sizes on the strength versus size power-law exponent.**

Power law exponent for polycrystals versus range of grain sizes studied as predicted from equation (1) and from four different experimental studies [5–8]. The initial dislocation density in the calculations was assumed to follow $\rho_0 = 10^{12}\,(1 + 10 \times 10^{-6}/D)$ µm.

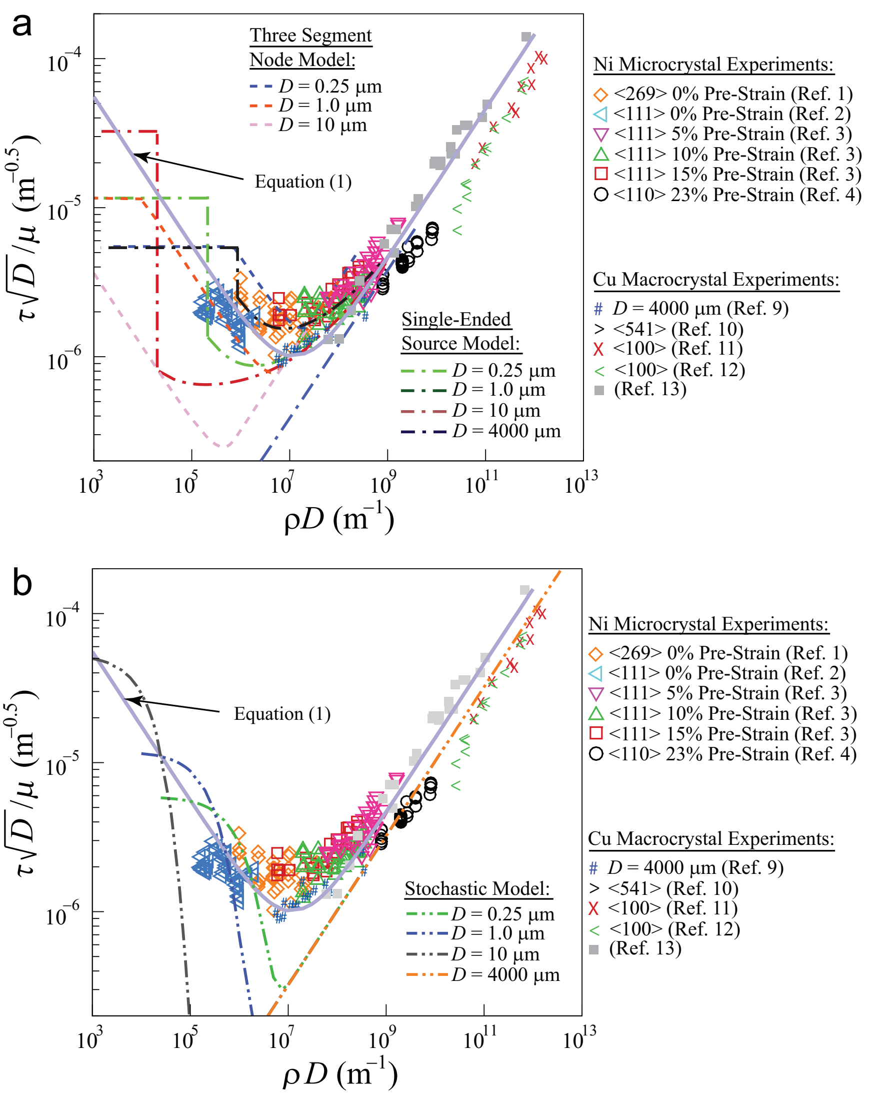

Supplementary Figure 3: **Comparison between existing models and the generalized size-dependent Taylor-strengthening law.**

Dimensionless resolved shear strength multiplied by the square root of the crystal diameter versus the initial dislocation density multiplied by the crystal diameter for Ni microcrystal [1–4] and Cu macrocrystal experiments [9–12]. Solid curve is Equation (1). In (a) the dashed lines are the "Three Segment Node Model" [13] and the dash-dot lines are the "Single-Ended Source Model" [14]; and in (b) the dash-dot-dot lines are the statistical model [15].

Supplementary Note 1: Physical Interpretation of the Generalized Size-Dependent Taylor-Strengthening Law

Equation (1) in the manuscript was expressed as follows:

$$\frac{\tau}{\mu} = \frac{\beta}{D\sqrt{\rho}} + \alpha b\sqrt{\rho} \tag{1}$$

The second term is clearly the traditional forest-strengthening term which states that the dislocation mean free path is inversely proportional to the square root of the dislocation density. This term is naturally obtained from the slope of the data in Figure 3 in the manuscript when $\alpha b\sqrt{\rho} >> \beta/D\sqrt{\rho}$. On the other hand, when $\alpha b\sqrt{\rho} << \beta/D\sqrt{\rho}$, the forest hardening term becomes negligible and the strength is mainly governed by the resolved shear strength required to activate the weakest dislocation-sources in the crystal.

The dislocation source strength is proportional to the inverse of the effective (or mean) source length, λ, which in turn must be bounded by the physical size of the crystal. Thus, equation (1) may be rewritten in the more familiar form of:

$$\frac{\tau}{\mu} = \frac{kb}{\lambda} \tag{2}$$

where k is a strength coefficient that is typically assumed to be between 0 and 1. By comparing equations (1) and (2), we find that the effective dislocation source length when $\alpha b\sqrt{\rho} << \beta/D\sqrt{\rho}$

should be given by:

$$\lambda = \frac{bD\sqrt{\rho}}{\beta} \tag{3}$$

while when $\alpha b\sqrt{\rho} >> \beta/D\sqrt{\rho}$ it would be given by

$$\lambda = \alpha/\sqrt{\rho} \tag{4}$$

where k was set to unity. These two equations give a relationship for the effective source length as a function of the crystal size and dislocation density. Equation (3) states that the effective dislocation source length increases proportionally with the crystal external dimensions, D, normalized by the mean free path $(1/\sqrt{\rho})$.

Supplementary Note 2: Effect of Dislocation Density and Range of Studied Crystal Sizes on the Power-Law Exponent

The effect of extrinsic and intrinsic size on the strength of single crystal and polycrystalline materials is typically expressed by the following experimentally determined power-law relationship:

$$\sigma = \sigma_0 + kD^{-n} \tag{5}$$

where σ is the strength of the crystal, σ_0 is the friction stress, D is the extrinsic size for single crystals or grain size for polycrystals, k is a fitting constant, and n is the fitted power-law exponent (between 0 and 1 for single crystals or 0 and 0.5 for polycrystals). Recently, the effect of pre-straining on the power-law exponent for single crystals has received some attention. However, questions regarding the effect of the range of studied crystal sizes, experimental scatter, and initial dislocation density are not fully addressed in many Hall-Petch related studies.

To address these questions, the power-law exponent, n, in equation (5), as computed for Ni based on equation (1) for different ranges of intrinsic and extrinsic crystal sizes, is shown in Supplementary Figure 1. The exponent computed from the current DDD simulations and from different experimental studies are also shown for comparison. For single crystals, the agreement between the analytical predictions, DDD simulation, and experiments is excellent. The slight deviations for some of the experimentally computed exponents at high dislocation densities can be attributed to the fact that the initial dislocation densities in the experiments are only approximations of the bulk crystal they were fabricated into. It is also clear that the predicted exponent depends on the range of crystal sizes investigated. For example, at a density of 10^{12} m^{-2} the exponent varies from 0.4 for crystals in the range $0.25 \leq D \leq 4000$ μm to 0.8 for crystals in the range $0.25 \leq D \leq 2.5$ μm.

In addition, for Ni polycrystals the analytically predicted exponents are shown in Supplementary Figure 1(b). The effect of the range of studied grain sizes is clear. Moreover, the maximum predicted exponent is $n = 0.5$, however, a few experimental studies suggest that the exponent can

be as large as $n = 1$. Such large exponent values have been suggested to be mainly associated with grain boundary strengthening [16]. Supplementary Figure 2 shows the power law exponent as a function of range of grain sizes studied as predicted from equation (1), and from four different experimental studies. The initial dislocation density in the calculations was assumed to be $\rho_0 = 10^{12}\,(1 + 10 \times 10^{-6}/D)$ µm. The agreement between the simulations and experiments is excellent with the only major deviation being the data from Meyers et al (2006) [8], which may be due to uncertainties in the initial dislocation density in the experiments.

Supplementary Note 3: Comparison Between Dislocation-Based Strengthening Models in the Literature and Experiments

Supplementary Figure 3 shows a comparison between the three-segment node model [13], the single-ended source model [14], the statistical model [15], the generalized size-dependent dislocation-based model shown in equation (1), and Ni microscale experiments on a $\tau\sqrt{D}/\mu$ versus ρD plot. One modification has been made to the statistical model proposed by Phani et al. (2013). In their model, they assumed a constant bulk strength independent of dislocation density. Instead, here the bulk stress is replaced by the traditional Taylor-strengthening law, $\sigma_b = \alpha\mu b\sqrt{\rho}$, where $\alpha = 0.4$. The material properties used for all analytical predictions are those of Ni. It is clear that previously proposed analytical models do not agree well with the experimental results. Experimental data and DDD simulations indicate that the results will fall on a single curve, which is captured correctly by our generalized size-dependent dislocation-based model (equation (1)). However, other analytical models show different curves for different crystal sizes and the magnitude of the stress at a certain

density deviate from experiments, especially for crystal sizes greater than 1 μm.

Supplementary References

1. Dimiduk, D., Uchic, M. & Parthasarathy, T. Size-affected single-slip behavior of pure nickel microcrystals. *Acta Mater.* **53**, 4065–4077 (2005).

2. Frick, C., Clark, B., Orso, S., Schneider, A. & Arzt, E. Size effect on strength and strain hardening of small-scale [111] nickel compression pillars. *Mater. Sci. Eng. A* **489**, 319–329 (2008).

3. Schneider, A. *et al.* Influence of bulk pre-straining on the size effect in nickel compression pillars. *Mater. Sci. Eng. A* **559**, 147 – 158 (2013).

4. El-Awady, J. *et al.* Pre-straining effects on the power-law scaling of size-dependent strengthening in Ni single crystals. *Scrtipta Mater.* **68**, 207–210 (2013).

5. Wang, Y. *et al.* Controlling factors in tensile deformation of nanocrystalline cobalt and nickel. *Phys. Rev. B* **85**, 014101 (2012).

6. Ebrahimi, F., Bourne, G., Kelly, M. & Matthews, T. Mechanical properties of nanocrystalline nickel produced by electrodeposition. *Nanostruct. Mater.* **11**, 343–350 (1999).

7. Thompson, A. Effect of grain size on work hardening in nickel. *Acta Metall.* **25**, 83–86 (1977).

8. Meyers, M., Mishra, A. & Benson, D. Mechanical properties of nanocrystalline materials. *Prog. Mater. Sci.* **51**, 427–556 (2006).

9. Hildebrand, H. The effect of the initial dislocation density on dislocation multiplication and work-hardening characteristics of copper single crystals. *Phys. Stat. Sol. (a)* **12**, 239–249 (1972).

10. Jackson, P. & Basinski, Z. Latent hardening and the flow stress in copper single crystals. *Can. J. Phys.* **45**, 707–735 (1967).

11. Ambrosi, P., Homeier, W. & Schwink, C. Measurement of dislocation density in [110] and [111] copper single crystals with high relative accuracy. *Scripta Metall.* **14**, 325–329 (1980).

12. van Drunen, G. & Saimoto, S. Deformation and recovery of [001] oriented copper crystals. *Acta Metall.* **19**, 213–221 (1971).

13. Johnson, L. & Ashby, M. The stress at which dislocations multiply in well-annealed metal crystals. *Acta Metall.* **16**, 219 – 225 (1968).

14. Parthasarathy, T., Rao, S., Dimiduk, D., Uchic, M. & Trinkle, D. Contribution to size effect of yield strength from the stochastics of dislocation source lengths in finite samples. *Scripta Mater.* **56**, 313–316 (2007).

15. Phani, P., Johanns, K., George, E. & Pharr, G. A simple stochastic model for yielding in specimens with limited number of dislocations. *Acta Mater.* **61**, 2489 – 2499 (2013).

16. Hutchison, M. & Pascoe, R. Grain-boundary strengthening in copper-base solid solutions. *Metal Sci. J.* **6**, 90–95 (1972).
